# Differential Expression Genes of the Head Kidney and Spleen in *Streptococcus iniae*-Infected East Asian Fourfinger Threadfin Fish (*Eleutheronema tetradactylum*)

**DOI:** 10.3390/ijms24043832

**Published:** 2023-02-14

**Authors:** Shun Maekawa, Pei-Chi Wang, Shih-Chu Chen

**Affiliations:** 1 Department of Veterinary Medicine, College of Veterinary Medicine, National Pingtung University of Science and Technology, Pingtung 91201, Taiwan; 2 International Degree Program of Ornamental Fish Technology and Aquatic Animal Health, International College, National Pingtung University of Science and Technology, Pingtung 91201, Taiwan; 3 General Research Service Centre, National Pingtung University of Science and Technology, Pingtung 91201, Taiwan

**Keywords:** *Streptococcus iniae*, RNA-seq, immune response, East Asian fourfinger threadfin fish (*Eleutheronema tetradactylum*), head kidney, spleen

## Abstract

*Streptococcus iniae* is a Gram-positive bacterium and is considered a harmful aquaculture pathogen worldwide. In this study, *S. iniae* strains were isolated from East Asian fourfinger threadfin fish (*Eleutheronema tetradactylum*) reared on a farm in Taiwan. A transcriptome analysis of the head kidney and spleen was performed in the fourfinger threadfin fish 1 day after infection using the Illumina HiSeq™ 4000 platform for RNA-seq to demonstrate the host immune mechanism against *S. iniae*. A total of 7333 genes based on the KEGG database were obtained after the de novo assembly of transcripts and functional annotations. Differentially expressed genes (DEGs) (2-fold difference) were calculated by comparing the *S. iniae* infection and phosphate-buffered saline control group gene expression levels in each tissue sample. We identified 1584 and 1981 differentially expressed genes in the head kidney and spleen, respectively. Based on Venn diagrams, 769 DEGs were commonly identified in both the head kidney and spleen, and 815 and 1212 DEGs were specific to the head kidney and spleen, respectively. The head-kidney-specific DEGs were enriched in ribosome biogenesis. The spleen-specific and common DEGs were found to be significantly enriched in immune-related pathways such as phagosome, Th1, and Th2 cell differentiation; complement and coagulation cascades; hematopoietic cell lineage; antigen processing and presentation; and cytokine–cytokine receptor interactions, based on the KEGG database. These pathways contribute to immune responses against *S. iniae* infection. Inflammatory cytokines (IL-1β, IL-6, IL-11, IL-12, IL-35, and TNF) and chemokines (CXCL8 and CXCL13) were upregulated in the head kidney and spleen. Neutrophil-related genes, including phagosomes, were upregulated post-infection in the spleen. Our results could offer a strategy for the treatment and prevention of *S. iniae* infection in fourfinger threadfin fish.

## 1. Introduction

*Streptococcus iniae* is a Gram-positive, β-hemolytic streptococcal bacteria and a main aquaculture pathogen infecting at least 27 different species of fresh- and saltwater teleosts [1,2]. *Streptococcus iniae* has also been identified as an infectious agent in humans, with transmission to humans caused by handling *S. iniae*-infected fish [3]. Although the types of symptoms of the disease vary depending on the fish species, *S. iniae*-infected fish suffer from meningitis and total ophthalmitis, resulting in high mortality rates [4]. *Streptococcus iniae* has been mainly found in North America, the Middle East, and the Asia-Pacific region. The most damaging impact of *S. iniae* has been observed in aquaculture, with estimated worldwide economic losses of over $100 million USD per year [5].

Understanding the mechanisms of host immune responses against pathogens is important for developing strategies for infectious disease treatment and prevention. There have been many studies on immune responses to *S. iniae* infection in various fish species, including tilapia (*Oreochromis* spp.) [6], rock bream (*Oplegnathus fasciatus*) [7,8], big-belly seahorse (*Hippocampus abdominalis*) [9], turbot (*Scophthalmus maximus* L.) [10], red sea bream (*Pagrus major*) [11], and zebrafish (*Danio rerio*) [12]. RNA sequencing (RNA-seq) using next-generation sequencing (NGS) has become a key technology in transcriptome analysis in the aquaculture field. The use of RNA-seq in aquaculture studies enables the investigation of organisms without reference genome sequences [13]. RNA-seq analyses have demonstrated the immune reactions in various aquaculture fish during various pathogenic infections [14]. We have previously revealed the diversity of immune-related genes in different fish species 1 day after bacterial infection via transcriptome profiling [14]. It is important to increase our knowledge of the immune mechanisms of different fish species against different pathogens. A transcriptome analysis was been performed on the spleen after *S. iniae* infection in tilapia using RNA-seq [15]. The differentially expressed genes (DEGs) that are upregulated after infection include those in several immune-related pathways, including pathogen attachment and recognition, cytoskeletal rearrangement, and immune activation or inflammation [15].

The fish species in Taiwan that is most susceptible to *S. iniae* infection is tilapia [16]. *Streptococcus iniae* was isolated and identified from East Asian fourfinger threadfin fish (*Eleutheronema tetradactylum*) in Pingtung, Taiwan, in 2018. East Asian fourfinger threadfin fish are an excellent food resource and are in demand in the domestic market (100–140 TWD/kg) because of their fast growth and remarkable meat quality [17]. However, the immune responses against *S. iniae* in this species have not been clearly elucidated. Therefore, the transcriptome of the head kidney and spleen (immune organs in teleosts) of fourfinger threadfin fish were examined 1 day after *S. iniae* infection to understand the immune mechanisms. Based on DEGs, tissue-specific and common immune-related pathways responding to *S. iniae* infection based on the Kyoto Encyclopedia of Genes and Genomes (KEGG) pathways were investigated. Our results of transcriptome sequences and DEGs could serve as valuable resources for further research and for devising effective strategies for the treatment and prevention of *S. iniae* infection in teleosts.

## 2. Results

### 2.1. Clean Reads and De Novo Assembly of Transcripts

A transcriptome analysis of the head kidney and spleen samples from both the infected and control groups was performed at 1 day post-infection (dpi) using an Illumina HiSeq™ 4000 platform. Over 30 million clean reads with high percentages of Q20 and Q30 were obtained in all samples after quality filtering and data trimming (Table S2). Totals of 37,655 and 39,965 unigenes were assembled as transcripts in the head kidney and spleen samples, respectively (Table S3).

### 2.2. Differentially Expressed Genes after S. iniae Infection and their Validation Using RT-qPCR

**Figure 1:**
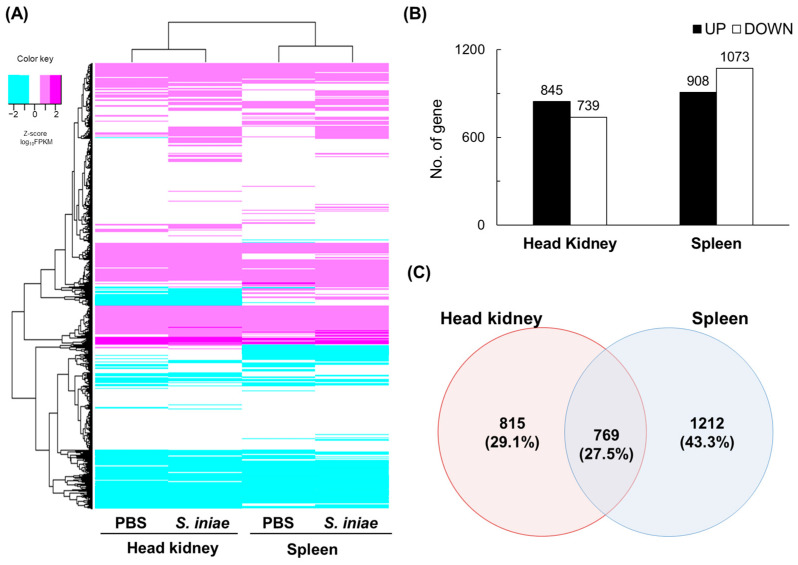
Gene expression profiles in a transcriptomic dataset. (**A**) Clustering by gene expression profiles in a transcriptomic dataset. Z-Scores were calculated using fragments per kilobase of transcripts per million mapped reads (FPKM) values and colored with magenta as the maximum and cyan as the minimum. The heat maps and clusters were produced using the gplots package in R software (version 4.2.2). (**B**) Numbers of differential expression genes (DEGs) in the head kidney and spleen in the *Streptococcus iniae*-infected and phosphate-buffered saline (PBS)-treated groups. (**C**) Venn diagrams showing overlaps of DEGs between the head kidney and spleen. The numbers indicate DEGs in each category.

The unigenes were clustered based on KEGG annotation prior to the determination of DEGs, and the mRNAs were detected from a total of 7333 *E. tetradactylum* genes. Genes and samples were hierarchically clustered using a heatmap based on the z-scored kilobase of transcripts per million mapped reads (FPKM) values. The heatmap revealed categorizations by tissue rather than by the presence or absence of infection (Figure 1A). The DEGs in each tissue sample were determined according to the relative expression levels of genes in the *S. iniae*-challenged group compared to those in the PBS groups, based on FPKM values. The total numbers of DEGs in the head kidney and spleen were 1584 (845 upregulated and 739 downregulated genes) and 1981 (908 upregulated and 1073 downregulated genes), respectively (Figure 1B). Based on the DEGs of the head kidney and spleen, overlapping and specific DEGs were identified. Venn diagrams (Figure 1C) showed that 769 DEGs were common to the head kidney and spleen. The numbers of specific DEGs in the head kidney and spleen were 815 and 1212, respectively.

**Figure 2:**
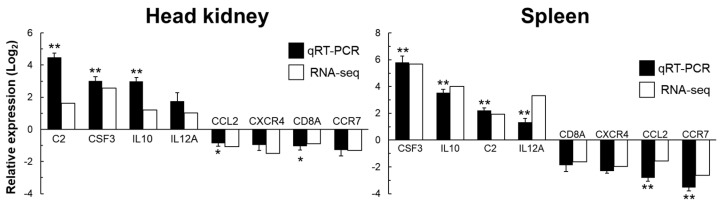
Validation of RNA-seq data by RT-qPCR. A gene expression level analysis in the head kidney and spleen from RNA sequencing (RNA-Seq) and reverse transcription quantitative real-time PCR (RT-qPCR) assays of fish infected with *Streptococcus iniae*. Total RNA samples from the head kidney and spleen were extracted, and the expression levels of each gene were determined using RT-qPCR assays. Expression levels of each target gene were normalized using HMBS as a control gene. The graph shows the relative gene expression levels of the *S. iniae*-infected and phosphate-buffered saline (PBS)-treated groups (*n* = 6, * *p* < 0.05, ** *p* < 0.01 compared to the PBS group).

An RT-qPCR analysis was performed to measure the relative expression levels of eight DEGs in the head kidney and spleen samples (Figure 2). The relative expression levels of each gene were consistent with the RNA-seq data. These results indicated that the transcriptome data were reliable.

### 2.3. Functional Enrichment Analysis of DEGs in the KEGG Pathways

A functional enrichment analysis of the DEGs was performed based on the KEGG pathway (Table 1) using the specific and common DEGs in the head kidney and spleen.

Specific DEGs in the head kidney were significantly assigned to two KEGG pathways: ribosome biogenesis in eukaryotes (23 genes, 2.82%) and RNA polymerase (11 genes, 1.35%). Spleen-specific DEGs were significantly assigned to 25 KEGG pathways. The immune-related DEGs were classified into seven pathways, including phagosome (29 genes, 2.39%), Th1 and Th2 cell differentiation (19 genes, 1.57%), complement and coagulation cascade (21 genes, 1.73%), Th17 cell differentiation (20 genes, 1.65%), hematopoietic cell lineage (17 genes, 1.40%), necroptosis (23 genes, 1.90%), and JAK-STAT signaling (23 genes, 1.90%) pathways. DEGs commonly found in the head kidney and spleen were significantly assigned to 28 KEGG pathways, including complement and coagulation cascade (18 genes, 2.34%), cytokine–cytokine receptor interaction (29 genes, 3.77%), hematopoietic cell lineage (13 genes, 1.69%), viral protein interaction with cytokines and cytokine receptor (12 genes, 1.56%), and IL-17 signaling (14 genes, 1.82%) pathways.

### 2.4. Specific Induction of Ribosome-Biogenesis-Related Genes in the Head Kidney

**Figure 3:**
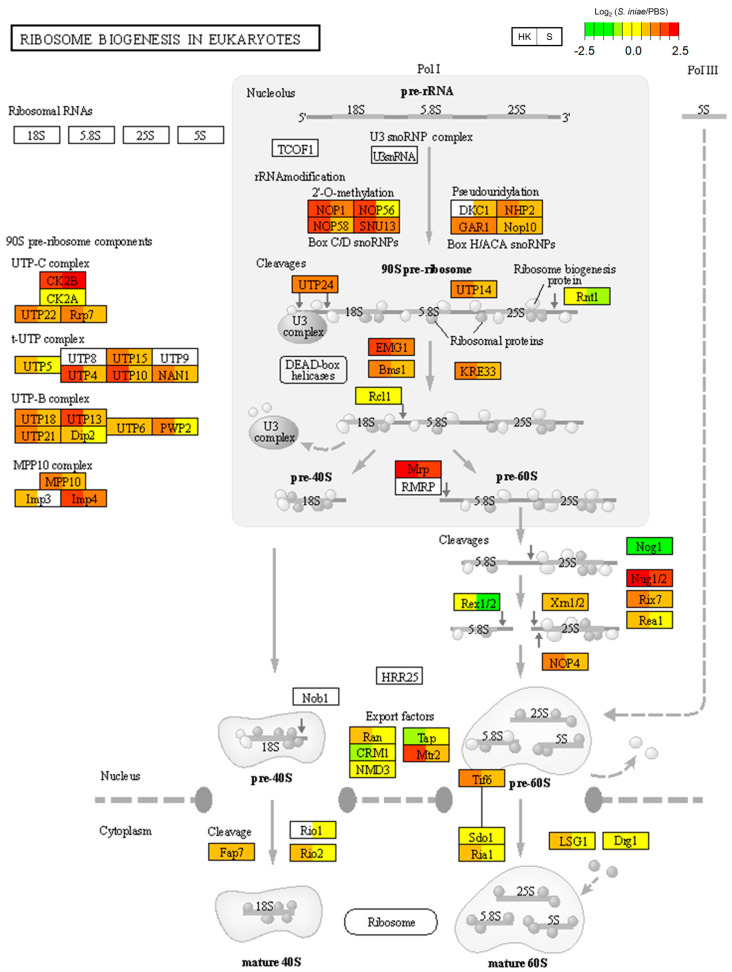
Ribosome biogenesis in eukaryotes in KEGG pathway maps (ko03008). Relative gene expression levels in the *Streptococcus iniae*-infected and phosphate-buffered saline (PBS)-treated groups are shown in the KEGG pathway map of ribosome biogenesis in eukaryotes (ko03008). The gene expression levels for the two groups, head kidney (HK) and spleen (S), are shown in each gene box. The higher expression levels are shown in red and the lower expression levels are shown in green. Undetected genes are shown in white.

The immune-related signaling pathways were investigated in the next step based on the KEGG enrichment analysis (Table 1). The KEGG pathway map was visualized using the gene expression data from our RNA-seq analysis using PathView [18]. Figure 3 shows a KEGG pathway map (ko03008) depicting ribosome biogenesis in eukaryotes, which was enriched due to kidney-specific DEGs (Table 1). *Streptococcus iniae* infection enhanced the expression of 90S pre-ribosomal components (UTPs), rRNA modification genes (NOP56, NOP58, NHP2), and cleavage genes (UTP14, Bms1, KRE33) in the head kidney.

### 2.5. Splenic DEGs Assigned to the KEGG Pathways

**Figure 4:**
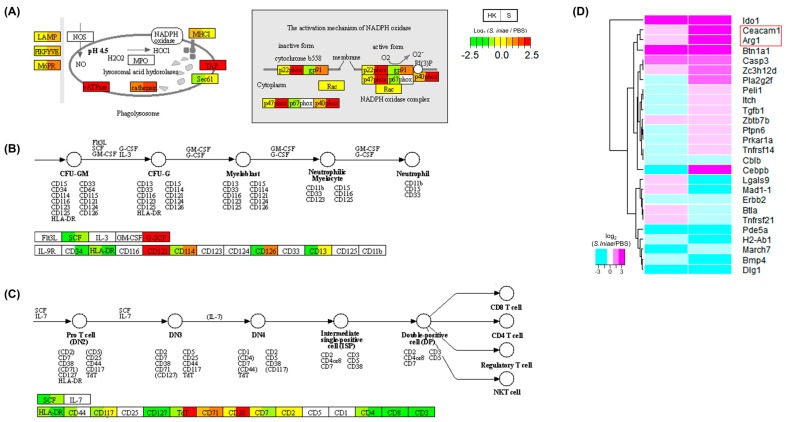
Spleen-specific pathway maps of phagosome and hematopoietic cell lineage in KEGG. Relative gene expression levels in the *Streptococcus iniae*-infected and phosphate-buffered saline (PBS)-treated groups are shown in these visualizations of (**A**) phagosomes (ko04145), focusing on phagolysosomes and the hematopoietic cell lineage (ko04640) pathway, neutrophils (**B**), and T-cells (**C**). The gene expression levels for the two groups, head kidney (HK) and spleen (S), are shown in each gene box. The higher expression levels of genes are shown in red, and lower expression levels are shown in green. Undetected genes are shown in white. (**D**) Clustering by gene expression profiles related to the negative regulation of T-cells based on the Gene Ontology database. Scores are colored on a log 2 scale (*S. iniae*-infected/PBS groups) with magenta as the maximum and cyan as the minimum. Heat maps and clustering were generated using the gplots package in R software.

Table 1 shows the splenic DEGs assigned to the KEGG pathways. Accordingly, the pathway map of the phagosome (ko04145) was investigated as one of the components of the immune response against bacterial infection (Figure 4A). Phagolysosome components, such as γATPase, TAP, and cathepsin, were highly expressed in both the head kidney and spleen after infection. Additionally, the pathway map showed enhancements of the NADPH-related genes (p22phox, p40phox, p47phox, gp91) and C-lectin receptors (MR, DCSIGN) in the spleen after infection. Next, the splenic DEGs enriched in the hematopoietic cell lineage pathway (ko04640) were investigated (Table 1). G-CSF and CD121 were highly expressed in the head kidney and spleen in the neutrophil lineage (Figure 4B) after infection. In addition, the expression levels of CD114 (G-CSF receptor) and CD126 were upregulated in the spleen after infection. Meanwhile, the mature T-cell markers of the T-cell lineage, including CD4, CD8, and CD3, were downregulated in the spleen after infection (Figure 4C). The categories of negative regulation in T cells based on Gene Ontology sequences, the negative regulation of activated T cell proliferation (GO:0046007), the negative regulation of CD4-positive, alpha-beta T cell proliferation (GO:2000562), the negative regulation of immature T cell proliferation (GO:0033088), the negative regulation of NK T cell proliferation (GO:0051141), and the negative regulation of T-cell proliferation (GO:0042130) were examined to identify the T-cell-related genes upregulated after *S. iniae* infection. Figure 4D shows the gene expression levels of negative regulators in T cells using a heat map. The expression levels of arginase 1 (Arg1) and carcinoembryonic antigen-related cell adhesion molecule 1 (Ceacam1) were found to be upregulated.

### 2.6. Common DEGs Enriched in Immune-Related KEGG Pathways

**Figure 5:**
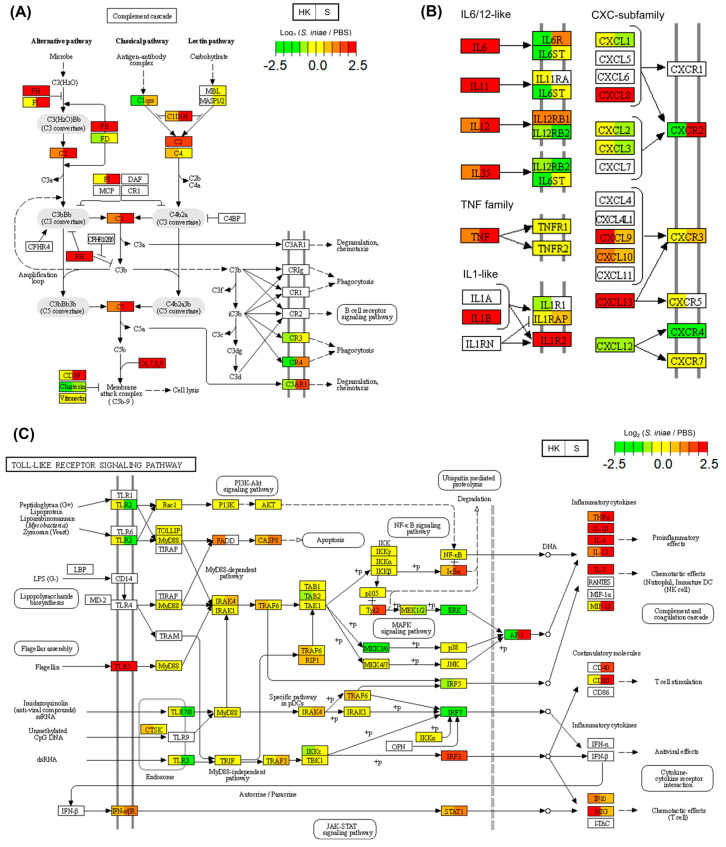
Pathway maps enriched by common differentially expressed genes (DEGs) between the head kidney and spleen after *Streptococcus iniae* infection. Relative gene expression levels in the *S. iniae*-infected and phosphate-buffered saline (PBS)-treated groups are shown in these visuals of complement and coagulation cascades (**A**), cytokine–cytokine receptor interactions (**B**), and Toll-like receptor signaling (**C**). The gene expression levels for the two groups, head kidney (HK) and spleen (S), are shown in each gene box. The lower expression levels of genes are shown in green, and the higher expression levels of genes are shown in red. Undetected genes are shown in white (see color legend in figure).

Figure 1C and Table 1 show the common DEGs enriched in the KEGG pathways. Figure 5A shows the complement cascade in the KEGG database (ko04610). The expression of C2, C3, C5, C7, C8A, C8B, and C9 genes was upregulated in the head kidney and spleen after infection. The C5AR1 gene expression in the spleen also increased after infection. Many immune-related genes were identified in the cytokine–cytokine receptor interaction pathway (ko04060) (Figure 5B). The results showed that the *S. iniae* infection induced the expression of inflammatory cytokines (IL-1β, IL-6, IL-11, IL-12, IL-35, and TNF) and chemokines (CXCL8 and CXCL13) in the head kidney and spleen. Additionally, the CXCL9 and CXCL10 levels in the head kidney increased after infection. CXCR4 was downregulated in the head kidney and spleen. The upstream pathways that induce inflammatory cytokines were identified based on the KEGG database. Figure 5C shows a Toll-like receptor signaling pathway map. *Streptococcus iniae* infection was found to induce the gene expression of TLR5 upstream of the inflammatory cytokines and chemokines in the head kidney and spleen.

**Figure 6:**
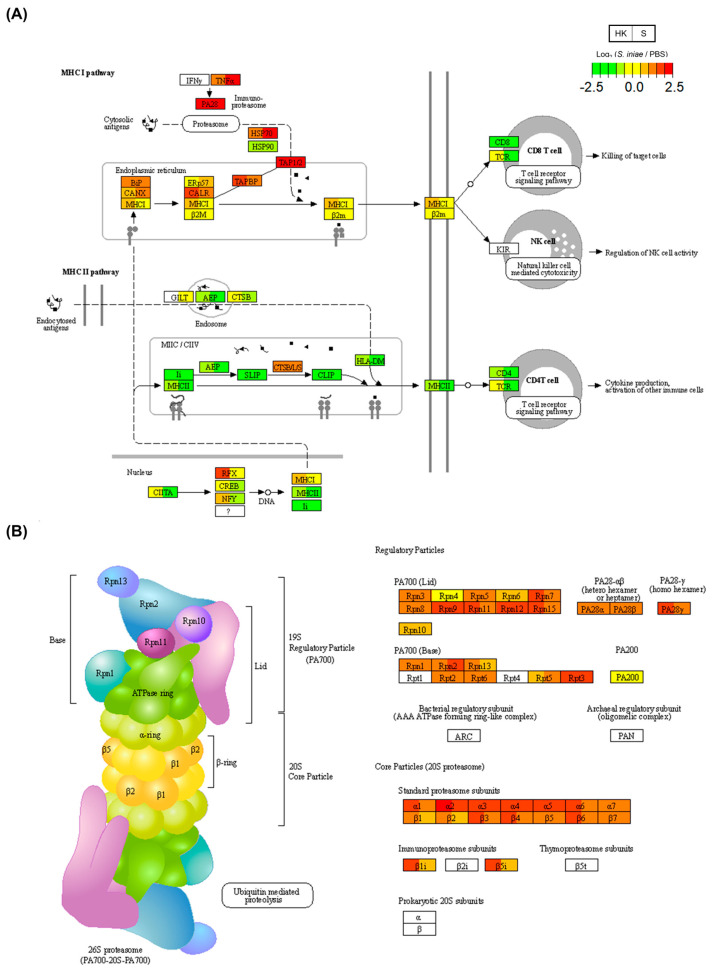
Antigen processing and presentation pathway map in the KEGG. Relative gene expression levels in the *Streptococcus iniae*-infected and phosphate-buffered saline (PBS)-treated groups are shown in these visualizations of (**A**) antigen processing and presentation (ko04612) and (**B**) proteasome pathways. The gene expression levels across four groups (K: kidney; S: spleen; 1: 1 day post-infection (dpi); 2: 2 dpi) are shown in each gene box. The lower expression levels of genes are shown in green, and the higher expression levels of genes are shown in red. Undetected genes are shown in white (see color legend in figure).

Finally, the common DEGs enriched in the antigen processing and presentation pathways were investigated (Figure 6A, Table 1). The MHC-I pathway-related genes (TNF, HSP70, TAP1/2, and TAPBP) were upregulated after infection in the head kidney and spleen compared to those of the MHC-II pathway. Additionally, the proteasome pathway is an important part of the MHC-I machinery that is required for antigen processing before its presentation. Table 1 shows that the proteasome pathway was enriched by common DEGs. Figure 6B shows the proteasome pathway in the KEGG, and the expressions of many proteasome genes were upregulated in the head kidney and spleen.

## 3. Discussion

The RNA-seq analysis of the head kidney and spleen of fourfinger threadfin fish (*Eleutheronema tetradactylum*) was performed 1 day post-infection with *S. iniae*, and a total of 7333 genes that were clustered by KEGG annotation were identified. Additionally, a total of 1584 and 1981 DEGs were identified in the head kidney and spleen, respectively. Tissue-specific and common immune-related genes responding to *S. iniae* infection were explored based on the information obtained on the DEGs assigned to the KEGG pathways.

Head-kidney-specific DEGs were enriched in only two KEGG pathways according to the KEGG database. Ribosome biogenesis in eukaryotes was investigated using KEGG pathway maps, and the head-kidney-specific enhancement of rRNA-related genes was identified (Figure 3). Ribosomes are universally important in organisms [19]. Several studies have shown an inverse relationship between immunity and ribosome biogenesis [20,21]. NOL-6 (also named UTP22) has been identified as a negative regulator of innate immunity in *Caenorhabditis elegans* [21]. *Streptococcus iniae* is commonly isolated from the brains and head kidneys of infected fish. It is speculated that the enhancement of ribosome-biogenesis-related genes in the head kidney after infection might decrease the immune defense and lead to a relatively higher titer of *S. iniae* in the head kidney.

Phagosomes play an important role in pathogen elimination in the innate immune system. Phagosome-related genes were upregulated in the spleen (Figure 4A). Additionally, G-CSF promotes neutrophil production and activation, and neutrophil markers were upregulated after infection. Neutrophils comprise the largest proportion of leukocytes in vertebrates and typically contribute to innate immunity by phagocytosis and the secretion of granule proteins [22,23]. The results of this study indicated that phagocytosis by neutrophils is one of the key functions against *S. iniae* infection in fourfinger threadfin fish. Meanwhile, T-cell-related genes were found to be downregulated in the spleen after infection (Figure 4C). The key factors that induce the negative regulation of T-cell-related genes were investigated using a gene ontology database. The spleen-specific upregulation of CEACAM1 and ARG1 genes was observed after infection (Figure 4D). L-arginine, which is essential for T-cell proliferation, is metabolized by arginase into L-ornithine and urea [24]. It has been reported that ARG1 is mainly expressed in neutrophils and inhibits T-cell proliferation and activation through L-arginine metabolism [25,26]. ARG can be induced after *Streptococcus dysgalactiae* infection with the downregulation of T-cell-related genes in cobia (*Rachycentron canadum*) [27]. Our results and other reports indicate the possibility that the expression of ARG in neutrophils causes T-cell downregulation in the spleen of fourfinger threadfin fish after *S. iniae* infection. Furthermore, the overexpression of CEACAM1 resulted in the decreased proliferation of T-cells in a mammalian study [28]. It is suggested that the upregulation of CEACAM1 inhibits T-cell proliferation in fourfinger threadfin fish after *S. iniae* infection.

The complement system is a major innate immune system in teleosts [29,30]. The expression of C2, C3, C5, C7, C8A, C8B, and C9 genes was found to be upregulated in the head kidney and spleen after infection in this study (Figure 5A). These upregulated genes lead to the membrane attack complex based on the KEGG pathway [31]. Therefore, the complement system is also an immune response against *S. iniae* in the fourfinger threadfin fish. Cytokine and chemokine gene expression was induced in the head kidney and spleen after *S. iniae* infection (Figure 5B). Additionally, TLR5 was upregulated as an upstream pathway of induced cytokines after infection (Figure 5C). There are other similar reports of increased TLR5 expression after *S. iniae* infection [32,33]. TLR5 binds flagellin and activates an innate immune response; however, flagellin genes of *S. iniae* have not been reported [32,33]. Thus, further studies are needed to elucidate the interaction between *S. iniae* infection and TLR5 signaling.

Finally, common DEGs were found to be enriched in the antigen processing and presentation pathways of the MHC-I machinery, leading to its enhancement (Figure 6A). In addition, the expression of many proteasome genes was upregulated in the head kidney and spleen. MHC-I presents foreign peptide fragments from pathogens to CD8+ T cells for adaptive immune responses in mammals [34]. MHC-I peptide fragment complexes in teleosts are functionally similar to those of mammals [35,36]. T-cell-related genes were found to be downregulated after *S. iniae* infection in this study (Figure 3C). Therefore, it is possible that T-cell downregulation leads to serious illness or mortality in fourfinger threadfin fish after *S. iniae* infection.

## 4. Materials and Methods

### 4.1. Animals

Healthy East Asian fourfinger threadfin fish (*Eleutheronema tetradactylum*) (body weight 40 ± 5 g) were used in this study. The fish were kept in aerated seawater (28 ± 1 °C) in an outdoor facility and fed commercial dry pellets corresponding to 3% of their total body weight. The fish were acclimatized for two weeks before the experiments.

### 4.2. Streptococcus Iniae Challenge

*Streptococcus iniae* (OT107005-201SI) was isolated from East Asian fourfinger threadfin fish reared on a farm in Taiwan. Before infection, 20 fish were anesthetized with 2-phenoxyethanol and intraperitoneally injected with a non-lethal dose of 1.0 × 10^3^ cfu *S. iniae* (Manuscript in preparation) suspended in 100 μL of phosphate-buffered saline (PBS). Another batch of 20 fish were injected with 100 μL of PBS to serve as the control group. At one day post-infection (24 hpi), the head kidneys and spleens were sampled from six individuals each from the infection and control groups.

### 4.3. Total RNA Extraction, Sequence Library Preparation, and Sequencing

The methods of preparation for the total RNA, cDNA libraries, and sequencing were performed as described previously [27,37]. In brief, total RNA samples were extracted from the head kidneys and spleens using an RNA reagent (Zymeset, Taipei, Taiwan). Two micrograms of total RNA from each of the six samples was pooled in each experimental group. The RNA integrity and quantity were assessed using an RNA Nano 6000 Assay Kit in the Bioanalyzer 2100 system based on the RNA Integrity Number (RIN) (Agilent Technologies, Santa Clara, CA, USA). Sequencing libraries were generated using an NEBNext^®^ Ultra TM RNA Library Prep Kit for Illumina^®^ (New England Biolabs, Ipswich, MA, USA) following the manufacturer’s recommendations. The sequencing was performed using the Illumina HiSeq™ 4000 platform (Illumina, Inc., San Diego, CA, USA), and 150 bp paired-end reads were generated at BIOTOOLS Co., Ltd. (Kaohsiung, Taiwan).

### 4.4. Reads Data Filtration and De Novo Transcriptome Assembly

Raw data (raw reads) in FASTQ format were first processed through in-house scripts [38]. Clean data (clean reads) were obtained by trimming reads containing adapters and removing poly N sequences and reads of low quality from the raw data in this step. The Q20, Q30, and GC contents of the clean data were calculated simultaneously [39]. Trinity (version 2.6.6) was used to perform the transcriptome assembly after filtering the read data [13] and then CORSET (version 4.6) software was used to remove redundancy from the Trinity results [40]. Finally, BUSCO (version 3.0.2) was used to perform a quantitative assessment of the expected gene content of the transcriptome (unigenes) [41].

### 4.5. Gene Function Annotation

All unigenes were functionally annotated using the following databases: NCBI non-redundant protein (NR), Clusters of Orthologous Groups (COGs), the Kyoto Encyclopedia of Genes and Genomes (KEGG) using Diamond (0.8.22) [42], NCBI nucleotide sequences (NT) using NCBI BLAST (version 2.9.0) [43], and Gene Ontology (GO) using Blast2GO (version v2.5.0) [44].

### 4.6. Differentially Expressed Genes and Functional Enrichment Analysis

Gene expression levels were estimated using RSEM software for each sample as follows: (1) clean data were mapped back onto the assembled transcriptome; (2) the read count for each gene was obtained from the mapping results. Expression data of each transcript were obtained and the values of fragments per kilobase of transcript per million mapped reads (FPKM) were calculated using RSEM (version 1.2.28) [45]. DEGs were determined by a 2-fold difference in expression levels between the *S. iniae* infection and PBS control groups using edgeR software [46,47]. DEGs were classified according to the KEGG classification [48] and *p*-values were calculated using hypergeometric tests for the enrichment analysis based on KEGG pathways. The false discovery rate (FDR) was then calculated for each *p*-value, and an FDR not larger than 0.05 was defined as significant enrichment. PathView was used to visualize the KEGG pathways with differential expression levels [18].

### 4.7. Real-Time Reverse Transcription Polymerase Chain Reaction

DNase I-treated total RNA (1 µg) was used for cDNA synthesis using iScript™ cDNA synthesis kits (Bio-Rad Laboratories, Inc., Hercules, CA, USA). Reverse transcription quantitative real-time PCR (RT-qPCR) was performed using an iQ™ SYBR^®^ Green Supermix (Bio-Rad Laboratories, Inc.) and a CFX Connect Real-Time PCR Detection System (Bio-Rad Laboratories, Inc.). The mean threshold cycle was used to measure the relative expression levels, and the expression levels of genes were normalized to that of the hydroxymethylbilane synthase (HMBS) gene. We selected HMBS as the internal control based on RNA-seq data in this study, which showed that the relative expression levels presented less than 2-fold differences in each group. Primers for detecting target genes were designed using Primer3 plus software [49] and are listed in Table S1. Student’s t-test was used to compare the *S. iniae* infection and PBS groups. The statistical significance value was set to *p* < 0.05.

## 5. Conclusions

An RNA-seq-based transcriptome analysis of fourfinger threadfin fish infected with *S. iniae* was performed. A total of 7333 genes based on KEGG annotation were obtained and specific and common DEGs in the head kidney and spleen were identified. An enrichment analysis using the DEGs revealed the immune-related pathways responding to *S. iniae* infection, including ribosome biogenesis, phagosome, TLR5, and cytokine pathways. These pathways contribute to antibacterial responses after *S. iniae* infection. These data could provide a deeper understanding of the immune system and protective strategies against *S. iniae* infection in fish.

## Figures and Tables

**Table 1:** Functional enrichment analysis results of KEGG pathways for specific and common differentially expressed genes (DEGs) in the head kidney and spleen.

ID	Pathway Name	Count	(%)	Q-Value
Head kidney-specific DEGs				
ko03008	Ribosome biogenesis in eukaryotes	23	(2.82)	3.82 × 10^−5^
ko03020	RNA polymerase	11	(1.35)	2.16 × 10^−2^
Spleen-specific DEGs				
ko04080	Neuroactive ligand-receptor interaction	53	(4.37)	8.22 × 10^−7^
ko04721	Synaptic vesicle cycle	21	(1.73)	1.03 × 10^−5^
ko05171	Coronavirus disease—COVID-19	53	(4.37)	2.53 × 10^−5^
ko05323	Rheumatoid arthritis	23	(1.90)	4.48 × 10^−5^
ko04145	Phagosome	29	(2.39)	2.76 × 10^−4^
ko04514	Cell adhesion molecules	27	(2.23)	5.01 × 10^−4^
ko04966	Collecting duct acid secretion	10	(0.83)	5.71 × 10^−4^
ko05340	Primary immunodeficiency	14	(1.16)	2.97 × 10^−3^
ko04658	Th1 and Th2 cell differentiation	19	(1.57)	5.44 × 10^−3^
ko04911	Insulin secretion	18	(1.49)	6.12 × 10^−3^
ko04610	Complement and coagulation cascades	21	(1.73)	7.12 × 10^−3^
ko03010	Ribosome	31	(2.56)	1.66 × 10^−2^
ko04961	Endocrine and other factor-regulated calcium reabsorption	12	(0.99)	1.57 × 10^−2^
ko04924	Renin secretion	16	(1.32)	2.54 × 10^−2^
ko04659	Th17 cell differentiation	20	(1.65)	2.56 × 10^−2^
ko04640	Hematopoietic cell lineage	17	(1.40)	3.07 × 10^−2^
ko05200	Pathways in cancer	77	(6.35)	3.07 × 10^−2^
ko05110	*Vibrio cholerae* infection	13	(1.07)	2.93 × 10^−2^
ko04921	Oxytocin signaling pathway	25	(2.06)	3.20 × 10^−2^
ko05120	Epithelial cell signaling in *Helicobacter pylori* infection	17	(1.40)	3.21 × 10^−2^
ko04217	Necroptosis	23	(1.90)	3.23 × 10^−2^
ko04630	JAK-STAT signaling pathway	23	(1.90)	3.08 × 10^−2^
ko00010	Glycolysis/Gluconeogenesis	13	(1.07)	3.20 × 10^−2^
ko04071	Sphingolipid signaling pathway	22	(1.82)	4.44 × 10^−2^
ko04725	Cholinergic synapse	19	(1.57)	4.98 × 10^−2^
Common DEGs				
ko03050	Proteasome	29	(3.77)	3.55 × 10^−17^
ko05144	Malaria	15	(1.95)	3.82 × 10^−6^
ko05146	Amoebiasis	20	(2.60)	3.22 × 10^−5^
ko05020	Prion disease	42	(5.46)	5.65 × 10^−5^
ko05017	Spinocerebellar ataxia	27	(3.51)	6.15 × 10^−5^
ko04610	Complement and coagulation cascades	18	(2.34)	1.40 × 10^−4^
ko05322	Systemic lupus erythematosus	13	(1.69)	2.32 × 10^−4^
ko05133	Pertussis	16	(2.08)	2.20 × 10^−4^
ko00970	Aminoacyl-tRNA biosynthesis	12	(1.56)	4.71 × 10^−4^
ko04060	Cytokine–cytokine receptor interaction	29	(3.77)	5.66 × 10^−4^
ko04080	Neuroactive ligand-receptor interaction	27	(3.51)	1.18 × 10^−3^
ko04612	Antigen processing and presentation	12	(1.56)	5.30 × 10^−3^
ko01110	Biosynthesis of secondary metabolites	47	(6.11)	1.03 × 10^−2^
ko05169	Epstein–Barr virus infection	28	(3.64)	1.05 × 10^−2^
ko05012	Parkinson disease	33	(4.29)	1.17 × 10^−2^
ko05142	Chagas disease	16	(2.08)	1.16 × 10^−2^
ko05150	*Staphylococcus aureus* infection	9	(1.17)	2.04 × 10^−2^
ko00260	Glycine, serine and threonine metabolism	10	(1.30)	2.03 × 10^−2^
ko05010	Alzheimer disease	41	(5.33)	2.10 × 10^−2^
ko05134	Legionellosis	11	(1.43)	2.35 × 10^−2^
ko04640	Hematopoietic cell lineage	13	(1.69)	3.57 × 10^−2^
ko00590	Arachidonic acid metabolism	8	(1.04)	3.87 × 10^−2^
ko04061	Viral protein interaction with cytokine and cytokine receptor	12	(1.56)	3.71 × 10^−2^
ko05323	Rheumatoid arthritis	12	(1.56)	3.56 × 10^−2^
ko05022	Pathways of neurodegeneration—multiple diseases	46	(5.98)	3.88 × 10^−2^
ko04657	IL-17 signaling pathway	14	(1.82)	4.34 × 10^−2^
ko04974	Protein digestion and absorption	11	(1.43)	4.41 × 10^−2^
ko05016	Huntington disease	34	(4.42)	4.25 × 10^−2^

## Data Availability

Data sharing not applicable as no datasets were generated or analyzed in this study.

## Supplementary Materials

The supporting information can be downloaded at: https://www.mdpi.com/article/10.3390/ijms24043832/s1.
