# Supplementary material for: Differential Expression Genes of the Head Kidney and Spleen in Streptococcus iniae-Infected East Asian Fourfinger Threadfin Fish (Eleutheronema tetradactylum)
Source: Int J Mol Sci. 2023 Feb 14;24(4):3832. doi: 10.3390/ijms24043832 (PMC9958670; doi:10.3390/ijms24043832)
Supplement: Supplementary file 1 [file ijms-24-03832-s001.zip › ijms-2160746-supplementary.pdf]

Supplemental Tables

Table S1 Sequences of primers used for quantitative real-time PCR.

Gene		Sequence (5'- 3')
C2	Forward	GCATATTCACACAGAGAGTCAGAGG
	Reverse	AGTCACATCAGTGGGCTGTTTTAG
CCL2	Forward	TGCTGTCTCCTGCCTCAACTC
	Reverse	TGGACTTGCGTGTTTGTAACCT
CCR7	Forward	TCGTTGTCCAAAACAGGACTATGT
	Reverse	TGCTGTGGTGGCTGTGTTC
CD8A	Forward	TCTGGTGATTCTGGTGTTTTATGC
	Reverse	TGTGGTTCTGTCCCTCTTTTACTTC
CSF3	Forward	CAGATGATGATGAGCTCACTGG
	Reverse	TTTTCGCACAGAGGTTTGAGG
CXCR4	Forward	CAGCCACACACCCACATAGA
	Reverse	AACAGCCAAGCCACAAGAAAG
HMBS	Forward	CTCTACAACGGACAAGAGC
	Reverse	GATCTTACTGGCCGTGAC
IL10	Forward	GAACAACATCTTGGAGTTTTACCTG
	Reverse	TGTCCTCGGTTTTGGTTATGG
IL12A	Forward	ACGAAACTGCTATCAAGTCC
	Reverse	AGGAGTGCTTCTTCTTGATG

Table S2. Summary of sequencing reads after filtering

Sample	Clean reads	Clean Bases	Q20 (%)	Q30 (%)	GC (%)
PBS_head kidney	32.5 M	9.7 G	97.7	93.6	49.7
*SI*_head kidney	35.1 M	10.5 G	97.7	93.6	49.8
PBS_spleen	39.6 M	11.9 G	97.6	93.4	50.0
*SI*_spleen	34.4 M	10.3 G	97.6	93.2	49.2

Clean data are read counts filtered from raw data; clean bases: base number of raw data after filtering, (number of clean reads) × (sequence length). Q20: percentage of bases whose Q Phred values are greater than 20, (number of bases with Q Phred value > 20) / (Number of total bases) ×100; Q30: percentage of bases whose Q Phred values are greater than 30, (number of bases with Q Phred value > 30) / (number of total bases) ×100; GC: percentage of G and C bases out of the number of total bases, (G&C base number) / (Total base number) ×100. PBS, phosphate-buffered saline; *SI*, *Streptococcus iniae*.

Table S3 Length distribution and quality matrix of *de novo* assembled unigenes

Sample	Min Length	Mean Length	Median Length	Max Length	N50	N90	Total Unigene
Kidney_Unigene	301	1567	848	25024	2781	590	37655
Spleen_Unigene	301	1574	858	23999	2804	594	39965

N50 and N90: weighted median statistics at which 50% and 90%, respectively, of the total length are contained in transcripts that are greater than or equal to this value.
